# Orientation Sessions Regarding Application of Artificial Intelligence in Obstetric Nursing: Its Effect on Maternity Students’ Knowledge and Attitude

**DOI:** 10.1155/nrp/2885750

**Published:** 2026-10-27

**Authors:** Eman Mostafa Sayed Ahmed, Wafaa Gomaa Abdallah Eliewa, Somaia Ragab Eid, Samah Osman Ali, Eman Said Abo El Ella Hassan

**Affiliations:** ^1^ Faculty of Nursing Maternal and Neonatal Health Nursing Department Ain Shams University Cairo Egypt asu.edu.eg; ^2^ Maternal and New Born Health Nursing Department Beni-Suef University Beni-Suef Egypt bsu.edu.eg; ^3^ Faculty of Nursing Community and Mental Health Nursing Department University of Tabuk Tabuk Saudi Arabia ut.edu.sa; ^4^ Faculty of Nursing Maternity and Child Health Nursing Department University of Tabuk Tabuk Saudi Arabia ut.edu.sa; ^5^ Faculty of Nursing Maternal and Newborn Health Nursing Department Cairo University Cairo Egypt cu.edu.eg

**Keywords:** artificial intelligence (AI), attitude, knowledge, maternity nursing students, orientation sessions

## Abstract

Artificial intelligence (AI) is transforming healthcare and nursing education globally. Despite its growing relevance, limited research has explored how orientation sessions influence nursing students’ knowledge and attitudes toward AI, particularly in maternity care settings in Egypt. This study evaluated the effect of structured orientation sessions on maternity nursing students’ knowledge and attitudes regarding AI applications in obstetric nursing at Ain Shams and Beni Suef universities. A quasiexperimental pre–post design was conducted among convenient sample of (786) third‐year maternity nursing students. Data were collected using a validated knowledge questionnaire and a Likert attitude scale before and after AI‐focused orientation sessions. Sessions included interactive lectures and culturally adapted educational materials. Statistical analyses assessed within‐ and between‐group differences. Preintervention, both groups demonstrated low‐to‐moderate knowledge and mixed attitudes toward AI. Postintervention, significant improvements were observed in knowledge and positive attitudes in both groups (*p* < 0.001). Ain Shams students showed a higher postintervention awareness level (58.8%) compared to Beni Suef (19.8%). Attitudinal shifts were also more pronounced among Ain Shams students across multiple domains, including AI’s role in education, clinical practice, and student assessment. The study concluded that the orientation sessions improved knowledge, enhanced attitudes toward AI in obstetric nursing, and produced significant differences between the two study settings. Integrating AI education into nursing curricula is essential to prepare future professionals for technology‐integrated healthcare environments. Further research is recommended to explore long‐term impacts and scalability across diverse educational settings.

## 1. Introduction

Artificial intelligence (AI) is a rapidly evolving field that has become an integral component of modern society and the fourth industrial revolution. It influences how individuals access information, communicate, and manage daily activities, and has emerged as a major technological, economic, and political force. Advances in computing power and data availability have transformed AI from a theoretical concept into practical applications that increasingly shape professional and personal lives [1, 2].

Globally, governments and educational institutions are investing in AI education to prepare future professionals for workforce demands. International initiatives, such as the European Union’s Digital Education Action Plan, along with national AI strategies in China, South Korea, and the United States, emphasize integrating AI into higher education curricula. These efforts aim to foster innovation, enhance digital competencies, and ensure students are prepared to use AI technologies responsibly and effectively [3].

The application of AI in education has gained increasing attention due to its potential to improve teaching and learning processes. AI‐supported educational tools can enhance clinical reasoning, decision‐making, and problem‐solving skills through personalized learning, simulation, and real‐time feedback. In nursing education, particularly maternity nursing, AI can support competency‐based learning while promoting awareness of ethical and professional responsibilities associated with AI use [4].

In healthcare, AI has demonstrated significant potential to improve prediction, diagnosis, and treatment outcomes. Evidence from highly cited studies in maternity healthcare indicates that AI technologies enhance diagnostic accuracy, support clinical decision‐making, and improve patient outcomes. Meta‐analytic findings show high diagnostic sensitivity of AI in maternity services, contributing to improved quality of life and treatment adherence [5].

The integration of AI into healthcare systems presents both opportunities and challenges for healthcare professionals. AI can transform clinical practice by increasing efficiency, reducing errors, and supporting evidence‐based decision‐making. Understanding and reporting AI’s role in clinical settings is essential for successful implementation and for equipping healthcare providers with the knowledge and skills required to utilize these technologies effectively [6].

In nursing practice, AI applications support repetitive and data‐intensive tasks, facilitate patient data management, and provide real‐time decision support. Systematic reviews highlight AI’s effectiveness in diagnosis, prognosis, and management of complex clinical conditions, including obstetric and gynecological care. These applications contribute to reducing administrative workload, improving patient safety, and enhancing the overall quality of nursing care [7, 8].

Maternity nursing students’ awareness and attitudes toward AI are critical for the successful adoption of these technologies in clinical practice. Digital and AI literacy are essential competencies for nursing graduates, as students’ perceptions may significantly influence their learning experiences, engagement, and future professional practice. Positive or negative attitudes toward AI may affect students’ willingness to integrate technology into maternity nursing care [9, 10].

Orientation sessions represent an effective strategy for introducing maternity nursing students to AI applications, clarifying expectations, and fostering positive attitudes toward technological integration. Well‐structured orientation programs that employ diverse educational methods can enhance students’ understanding, engagement, and readiness to apply AI in clinical contexts [11].

AI is increasingly being introduced into obstetric nursing research in Egypt, especially in fetal monitoring, clinical decision support, and maternal health education. Recent studies in Egypt have highlighted the growing role of AI in obstetric and maternity nursing practice and education. AI technologies are increasingly being integrated into fetal monitoring, clinical decision‐making, nursing education, and patient care to improve maternal and neonatal outcomes. A study conducted at Sohag University Hospital reported that educational guidelines significantly improved maternity nurses’ knowledge and attitudes regarding AI applications in nursing practice, emphasizing the importance of continuous training programs for nurses [12]. Similarly, a study conducted at Minia University Hospital found that instructional sessions about AI‐assisted fetal monitoring positively enhanced nurses’ perspectives and attitudes toward the use of AI in maternity units [13].

In addition, AI‐based educational tools such as chatbots have shown promising results in maternity nursing education. A recent study at Mansoura University demonstrated that using an AI chatbot improved maternity nursing students’ performance and understanding of electronic fetal monitoring [14]. These findings indicate that AI applications are becoming an important component in the development of obstetric nursing practice and education in Egypt.

Despite the growing global emphasis on AI in healthcare and education, limited research has examined the impact of orientation sessions on maternity nursing students’ awareness and attitudes toward AI, particularly in Egypt. Therefore, this study aims to evaluate the effect of an orientation session on maternity nursing students’ knowledge and attitudes regarding the application of AI in maternity nursing practice [15].

### 1.1. Aim of the Study

This study aims to evaluate the effect of orientation sessions on maternity nursing students’ knowledge and attitudes toward the application of AI at the Faculty of Nursing, Ain Shams University, and the Faculty of Nursing, Beni Suef University.

### 1.2. Specific Objectives

- To assess maternity nursing students’ levels of knowledge regarding the application of AI in obstetric nursing.
- To assess maternity nursing students’ attitudes toward the application of AI in obstetric nursing.
- To design and implement orientation sessions focused on the application of AI in obstetric nursing.
- To evaluate the effectiveness of the orientation sessions in enhancing maternity nursing students’ knowledge and attitudes regarding AI.
- To compare the effects of the orientation sessions on maternity nursing students at the Faculty of Nursing, Ain Shams University, and the Faculty of Nursing, Beni Suef University.

### 1.3. Research Hypotheses

 
**H**_**1**_ **:** There will be a statistically significant improvement in maternity nursing students’ knowledge regarding the application of AI in obstetric nursing after attending the orientation sessions compared to their preintervention level.
 
**H**_**2**_ **:** There will be a statistically significant improvement in maternity nursing students’ attitudes toward the application of AI in obstetric nursing after attending the orientation sessions compared to their preintervention level.
 
**H**_**3**_ **:** There will be a statistically significant difference in the postintervention knowledge and attitude scores of maternity nursing students between Ain Shams University and Beni Suef University following the orientation sessions.

## 2. Methods

A quasiexperimental pre‐ to postintervention design was used. The design enabled comparison of changes in knowledge and attitude scores within each university and between the two study settings, thereby allowing assessment of the effectiveness of the intervention. The current study was conducted on third‐year maternity nursing students at the Faculty of Nursing—Ain Shams University and Beni Suef University, during the 2^nd^ semester of 2024/2025.

The study was conducted at Ain Shams and Beni Suef universities because they are major governmental nursing institutions representing different geographical regions in Egypt. Both universities provide maternity nursing education to a large number of students and had no prior formal AI orientation programs, making them appropriate settings for evaluating the effectiveness of structured orientation sessions.

### 2.1. Subjects

A convenient sample of (786) all available maternity nursing students enrolled in the 3rd‐year academic level at the Faculty of Nursing, Ain Shams University, and the Faculty of Nursing, Beni Suef University, during the 2^nd^ semester academic year 2024–2025. The sample was distributed between the two universities as follows: Beni Suef University (*n* = 400) and Ain Shams University (*n* = 386). Third‐year students were selected because they were the students studying the Maternity Nursing course. The required sample size was calculated to detect a statistically significant difference in mean knowledge and attitude scores before and after the orientation sessions using the following equation for comparing two related means (paired measurements):

(1) n=Zα/2+Zβ×σd2

Where:

• *n* = required sample size
• Zα/2 = standard normal deviate at 95% confidence level (1.96)
• Zβ = standard normal deviate corresponding to 80% power (0.84)
• *σ* = pooled standard deviation of awareness/attitude scores (estimated from a pilot study or previous literature)
• *d* = minimum expected mean difference between pre‐ and postintervention scores

Based on an assumed moderate effect size, a confidence level of 95%, and a study power of 80%, the calculated minimum sample size was increased to account for potential nonresponse and incomplete data. Accordingly, a total convenient sample of 786 third‐year maternity nursing students from Ain Shams and Beni Suef universities was included in the study.

### 2.2. Tools of Data Collection

The current study data were collected using two tools as follows:

Tool (I): Self‐Administered Maternity Students Artificial Intelligence Knowledge Questionnaire: This tool was developed by the researchers based on a review of the literature [7, 16–18]. It consists of two parts:

Part 1: Nursing student characteristics such as age, gender, marital status, level of education, years of nursing experience, previous training, or formal education regarding AI.

Part 2: Maternity students’ knowledge regarding the application of AI in the nursing field, e.g., “Do you know what’s the meaning of AI is?” etc. This part was developed by [19]and modified by the researcher to assess the knowledge of maternity students regarding the application of AI. It consists of eight questions.

The scoring system is a three‐point response ranging from *correct answer* (3), *the incomplete answer* (2), and *incorrect or I don’t know* (1) to measure the level of maternity students’ knowledge regarding the applications of AI in the nursing field. The maximum possible total score is 8–24. A higher score is indicative of a higher level of knowledge. The knowledge level is considered good/high if the percent score is more than 75%, moderate if the percent score ranges from 60 to ≤ 75%, and low if the percent score is less than 60%.

Tool (II): Likert Attitude Scale: This tool developed by [20] aims to measure nursing students’ attitudes toward AI and contains 13 elements.

The scoring system is a three‐point Likert scale: Every question has three responses ranging from *agree* (3), *neutral* (2), and *disagree* (1) to assess maternity students’ attitudes regarding the application of AI in the nursing field.

Attitude score ranges from 13 to 39: Scores between 13 and 31 indicate a negative attitude, and scores between 32 and 39 indicate a positive attitude.

Content validity: A panel of five experts in maternal and neonatal health nursing reviewed the tool for validity. The tool assessed clarity, comprehensiveness, simplicity, understanding, and applicability. Modifications, including rephrasing and adding or omitting statements, were performed according to the jury’s opinions.

Reliability: The internal consistency was measured to identify the extent to which the items of the tools measured the same concept and correlated with each other using the *r* test. It was done by calculating Cronbach’s alpha test and was estimated as (0.85) for Tool (I) and (0.90) for Tool (II).

### 2.3. Operational Designs

Preparatory phase: The current and international‐related literature using books, periodicals, journals, magazines, and the internet will be reviewed by the researcher to be more acquainted with the topic and with the process of tool design.

### 2.4. Ethical Considerations

Prior to the commencement of the study, ethical approval was obtained from the Scientific Research and Ethics Committee of the Faculty of Nursing, Beni Suef University (2503202539).

The Research Ethics Committee of Beni Suef University served as the primary approving body for the study protocol. This approval was subsequently submitted to Ain Shams University, where permission to collect data was granted after the faculty administration reviewed and accepted the ethical clearance issued by Beni Suef University. Data collection was conducted following the acquisition of all necessary administrative approvals from the relevant faculty authorities.

The study involved minimal‐risk procedures, including an educational intervention and the administration of an anonymous questionnaire. No invasive or clinical procedures were undertaken.

Prior to data collection, the researchers clearly explained the aim and objectives of the study to the participating maternity students. Written informed consent was obtained from each participant after providing a simple and comprehensive explanation of the study. Confidentiality and anonymity were ensured through the use of coded questionnaires. Participation was entirely voluntary, and participants had the right to withdraw from the study at any time without any consequences. All collected data were used solely for scientific research purposes.

### 2.5. Administrative Procedures

Official permission was obtained through the submission of a formal letter issued by the Dean of the Faculty of Nursing, Ain Shams University and Beni Suef University, to collect the necessary data for the current study after providing a brief explanation of the study purpose and its expected outcomes.

### 2.6. Pilot Study

A pilot study was conducted on 10% of the maternity students to evaluate the applicability and clarity of the study tools and to estimate the time required for data collection. Necessary modifications were made based on the results of the pilot study.

### 2.7. Fieldwork

The fieldwork was carried out over a period of three months (begin from January 2025 end with March 2025) during the second academic semester of the academic year 2024–2025. The study was conducted in two settings: the Faculty of Nursing, Ain Shams University, and the Faculty of Nursing, Beni Suef University. The fieldwork was implemented through the following phases:

Phase I: Preparation and Orientation: It was conducted during the first two weeks of January 2025. At the beginning of the study, orientation sessions were conducted for maternity nursing students at both faculties. These sessions were held in the main lecture halls during scheduled lecture times. The researchers explained the aim of the study, its significance, study procedures, and the nature of students’ participation. Ethical considerations, including voluntary participation, confidentiality, and the right to withdraw at any time, were clearly explained. Each session lasted between 45 and 60 min. Written informed consent was obtained from students who agreed to participate in the study.

Phase II: Preintervention Assessment: Following the orientation sessions, data collection was conducted online using Google Forms (https://forms.gle/NVedz5NhwZMqJJjMA) during the second and third weeks of January 2025 to ensure ease of access and uniformity across both study settings. Official online communication channels (e.g., WhatsApp groups) were created for participating students from both universities. A Google Form link was distributed, which included the preintervention assessment tools: Tool I (Maternity Students’ Artificial Intelligence Knowledge) and Tool II (Students’ Attitude toward the Application of Artificial Intelligence in the Nursing Field). Students were instructed to complete the questionnaires individually. Responses were automatically submitted to the researchers’ official academic email addresses.

Phase III: Intervention Implementation: After completion of the pretest assessment, the intervention was implemented from February to early March 2025 in the form of structured orientation sessions regarding the application of AI in obstetric nursing. Supportive educational materials were developed by the researchers in the Arabic language to ensure clarity and cultural appropriateness. The session content included the concept of AI, types of AI used in healthcare, advantages and disadvantages of AI in nursing, challenges and opportunities of AI in nursing, limitations of AI, ethical considerations related to AI use, World Health Organization (WHO) guiding principles for AI use in the maternity field, and maternity nursing roles in the era of AI. Each session lasted approximately 30–45 min.

Due to the large number of participants, the intervention could not be conducted for all students simultaneously. Therefore, students were divided into five main groups, each further subdivided into smaller subgroups.

Phase IV: Postintervention Evaluation: Evaluation was conducted immediately in March 2025 after completion of the orientation sessions to assess students’ knowledge and attitudes toward AI using the same assessment tools (Tool I and Tool II) conducted online using Google Forms https://docs.google.com/forms/d/e/1FAIpQLSel2yt_GehQ056lzBvejNXotWtu4Q0bJiTXAhrPMr_RtRTWBg/viewform?usp=preview.

### 2.8. Statistical Design

The collected data were organized, tabulated, analyzed, and presented in tables and graphs. Descriptive statistics, including means, standard deviations, and percentages, were calculated. Appropriate statistical tests were used to determine the significance of the obtained results.

## 3. Results

As shown in Table 1, the age distribution was similar across both student groups, with 76.4% of Ain Shams students and 75.3% of Beni Suef students aged between 18 and 20 years. The mean age was comparable: 19.7 ± 1.5 years for Ain Shams students and 19.7 ± 1.4 years for Beni Suef students.

**TABLE 1:** General characteristics of Ain Shams and Beni Suef students.

	Ain Shams (*n* = 386)		Beni Suef (*n* = 400)		Chi‐square	
	*N*	%	*N*	%	*X* ^2^	*p*
Age (Years)						
18–20	295	76.4	301	75.3		
21–23	91	23.6	99	24.8	0.148	0.701
Mean ± SD	19.7 ± 1.5		19.7 ± 1.4		0.208	0.835
Gender						
Male	128	33.2	122	30.5		
Female	258	66.8	278	69.5	0.641	0.423
Residence						
Rural	119	30.8	108	27.0		
Urban	267	69.2	292	73.0	1.402	0.236
Income						
Sufficient	231	59.8	226	56.5		
Insufficient	21	5.4	34	8.5		
Barely sufficient	134	34.7	140	35.0	3.010	0.222
Have you attended any training courses related to computer programming?						
Yes	29	7.5	28	7.0		
No	357	92.5	372	93.0	0.077	0.782
Have you received any educational sessions about artificial intelligence?						
Yes	50	13.0	56	14.0		
No	336	87.0	344	86.0	0.184	0.668

In terms of gender, Table 1 revealed that 66.8% of Ain Shams students and 69.5% of Beni Suef students were female. Additionally, a higher proportion of students from Beni Suef 73.0% reported living in urban areas, compared to 69.2% of students from Ain Shams.

Additionally, regarding income, Table 1 reported that 59.8% of Ain Shams students and 56.5% of Beni Suef students reported sufficient income, while the remaining percentages reported insufficient income. Regarding computer programming training courses, 92.5% of Ain Shams students and 93.0% of Beni Suef students had not attended any related training courses. Similarly, 87.0% of Ain Shams students and 86.0% of Beni Suef students reported that they had not received any educational sessions about AI.

No statistically significant differences were found between the two groups regarding these variables (*p* > 0.05).

Figure 1 illustrates that the most common sources of information about AI among students were scientific conferences and seminars, reported by 30.5% of the Beni Suef student group and 27.3% of the Ain Shams student group. This was followed by browsing the internet and social media, cited by 25.3% and 24.1% of students from the respective groups.

**FIGURE 1:**
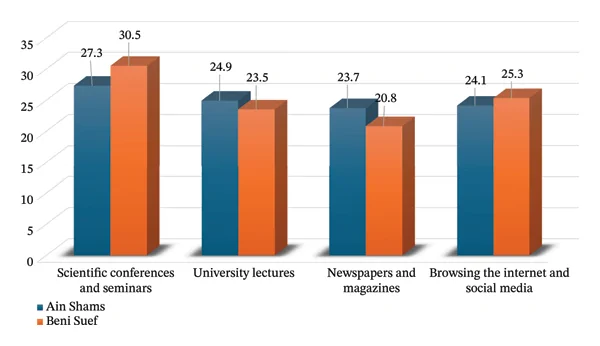
Students’ sources of information about artificial intelligence.

Table 2 reveals statistically significant differences and improvements in all items related to knowledge about AI following the educational orientation sessions in both groups.

**TABLE 2:** Comparison of knowledge levels within groups (pre‐ to postcomparisons for Ain Shams students and Beni Suef students, respectively) and between groups (Ain Shams vs. Beni Suef students).

	Pre–intervention						Post–intervention						(*p*‐value) between groups				(*p*‐value) within groups (pre vs. post)	
	Incorrect		Incomplete		Correct		Incorrect		Incomplete		Correct		Pre. vs pre		Post. vs post			
	*n*	%	*N*	%	*N*	%	*n*	%	*n*	%	*N*	%	*X* ^2^	*p*	*X* ^2^	*p*	*X* ^2^	*p*
Do you know what artificial intelligence is?																		
Ain Shams	132	34.2	138	35.8	116	30.1	61	15.8	76	19.7	249	64.5	0.218	0.897	35.695	< 0.001^∗∗^	92.545	< 0.001^∗∗^
Beni Suef	131	32.8	144	36.0	125	31.3	112	28.0	114	28.5	174	43.5					13.004	0.002^∗^
What are the advantages of using artificial intelligence applications in gynecology nursing?																		
Ain Shams	126	32.6	131	33.9	129	33.4	71	18.4	73	18.9	242	62.7	2.366	0.306	21.588	< 0.001^∗∗^	66.263	< 0.001^∗∗^
Beni Suef	128	32.0	155	38.8	117	29.3	101	25.3	114	28.5	185	46.3					24.744	< 0.001^∗∗^
What are the disadvantages of applying artificial intelligence in gynecology nursing?																		
Ain Shams	121	31.3	161	41.7	104	26.9	64	16.6	69	17.9	253	65.5	3.847	0.146	41.100	< 0.001^∗∗^	116.550	< 0.001^∗∗^
Beni Suef	136	34.0	140	35.0	124	31.0	112	28.0	117	29.3	171	42.8					11.869	0.003^∗^
What is the potential future role of artificial intelligence technology in gynecology nursing education?																		
Ain Shams	137	35.5	136	35.2	113	29.3	61	15.8	58	15.0	267	69.2	0.544	0.762	49.769	< 0.001^∗∗^	122.943	< 0.001^∗∗^
Beni Suef	143	35.8	132	33.0	125	31.3	100	25.0	122	30.5	178	44.5					17.273	< 0.001^∗∗^
What are the challenges facing the application of artificial intelligence technology in gynecology nursing education?																		
Ain Shams	132	34.2	142	36.8	112	29.0	71	18.4	59	15.3	256	66.3	0.774	0.679	24.418	< 0.001^∗∗^	108.952	< 0.001^∗∗^
Beni Suef	139	34.8	136	34.0	125	31.3	105	26.3	99	24.8	196	49.0					26.267	< 0.001^∗∗^
Do you have information on the various applications of artificial intelligence in healthcare (nursing)?																		
Ain Shams	136	35.2	138	35.8	112	29.0	72	18.7	67	17.4	247	64.0	0.636	0.728	37.621	< 0.001^∗∗^	95.049	< 0.001^∗∗^
Beni Suef	135	33.8	154	38.5	111	27.8	112	28.0	119	29.8	169	42.3					18.643	< 0.001^∗∗^
What is the role of artificial intelligence in healthcare?																		
Ain Shams	130	33.7	132	34.2	124	32.1	55	14.2	82	21.2	249	64.5	1.749	0.417	27.438	< 0.001^∗∗^	83.978	< 0.001^∗∗^
Beni Suef	138	34.5	150	37.5	112	28.0	98	24.5	117	29.3	185	46.3					28.801	< 0.001^∗∗^
What skills are required to develop gynecology nursing when using artificial intelligence technology?																		
Ain Shams	123	31.9	147	38.1	116	30.1	63	16.3	79	20.5	244	63.2	0.035	0.983	25.195	< 0.001^∗∗^	85.326	< 0.001^∗∗^
Beni Suef	125	31.3	154	38.5	121	30.3	104	26.0	114	28.5	182	45.5					20.176	< 0.001^∗∗^

^∗^ *p* < 0.05.

^∗∗^ *p* < 0.001.

Figure 2 shows differences in the levels of knowledge between the pre‐ and posteducational orientation sessions in both groups, while the higher knowledge level in the Ain Shams student group with 58.8% in compared with 19.8% in the Beni Suef student group.

**FIGURE 2:**
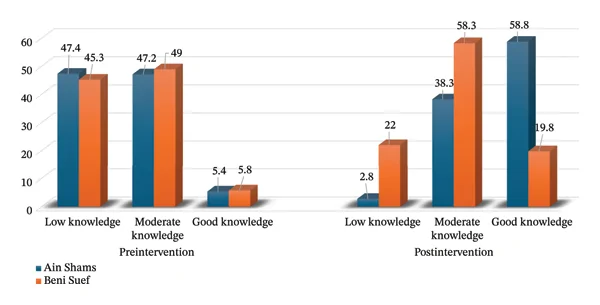
Comparison of students’ knowledge levels regarding artificial intelligence between Ain Shams and Beni Suef universities pre‐ and postintervention.

Table **3** reveals a highly statistically significant difference (*p* < 0.001) and an improvement in the students’ attitude mean scores in both groups following the educational orientation session on AI, compared to before the session.

**TABLE 3:** Comparison of attitude levels within groups (pre‐ and postintervention) and between groups (Ain Shams vs. Beni Suef students).

	Preintervention						Postintervention						(*p*‐value) between groups				(*p*‐value) within group (pre vs. post)	
	*Disagree*		*Neutral*		*Agree*		*Disagree*		*Neutral*		*Agree*		Pre vs pre		Post vs post			
	*N*	%	*N*	%	*n*	%	*N*	%	*n*	%	*N*	%	*X* ^2^	*p*	*X* ^2^	*p*	*X* ^2^	*p*
Do you support the application of artificial intelligence in the field of obstetrics and gynecology?																		
Ain Shams	121	31.3	93	24.1	172	44.6	62	16.1	76	19.7	248	64.2	1.794	0.408	13.344	< 0.001^∗∗^	34.484	< 0.001^∗∗^
Beni Suef	129	32.3	110	27.5	161	40.3	100	25.0	91	22.8	209	52.3					11.696	0.003^∗^
The development of artificial intelligence supports/assists obstetric nurses in acquiring new knowledge and skills.																		
Ain Shams	102	26.4	143	37.0	141	36.5	59	15.3	86	22.3	241	62.4	1.811	0.404	14.668	< 0.001^∗∗^	51.850	< 0.001^∗∗^
Beni Suef	115	28.7	130	32.5	155	38.8	95	23.8	107	26.8	198	49.5					9.375	0.009^∗^
Artificial intelligence has a positive impact on students’ academic performance.																		
Ain Shams	120	31.1	116	30.1	150	38.9	60	15.5	74	19.2	252	65.3	0.630	0.730	17.640	< 0.001^∗∗^	55.165	< 0.001^∗∗^
Beni Suef	114	28.5	125	31.3	161	40.3	102	25.5	93	23.3	205	51.2					10.654	0.005^∗^
Artificial intelligence should be integrated into educational curricula.																		
Ain Shams	125	32.4	117	30.3	144	37.3	66	17.1	73	18.9	247	64.0	1.961	0.375	16.471	< 0.001^∗∗^	55.548	< 0.001^∗∗^
Beni Suef	124	31.0	108	27.0	168	42.0	101	25.3	100	25.0	199	49.8					5.277	0.071
Artificial intelligence should be used as a tool for student assessment.																		
Ain Shams	119	30.8	113	29.3	154	39.9	64	16.6	65	16.8	257	66.6	0.344	0.842	31.185	< 0.001^∗∗^	55.287	< 0.001^∗∗^
Beni Suef	124	31.0	110	27.5	166	41.5	114	28.5	98	24.5	188	47.0					2.480	0.289
The use of artificial intelligence will improve maternal and fetal healthcare practices.																		
Ain Shams	129	33.4	101	26.2	156	40.4	75	19.4	59	15.3	252	65.3	3.798	0.150	27.178	< 0.001^∗∗^	47.907	< 0.001^∗∗^
Beni Suef	122	30.5	130	32.5	148	37.0	101	25.3	109	27.3	190	47.5					9.042	0.011^∗^
It is a tool that assists specialists in providing maternal and fetal healthcare.																		
Ain Shams	115	29.8	108	28.0	163	42.2	75	19.4	75	19.4	236	61.1	0.116	0.944	12.699	0.002^∗^	27.728	< 0.001^∗∗^
Beni Suef	116	29.0	116	29.0	168	42.0	80	20.0	119	29.8	201	50.2					9.602	0.008^∗^
Artificial intelligence assists in responding to emergencies.																		
Ain Shams	121	31.3	110	28.5	155	40.2	66	17.1	64	16.6	256	66.3	0.627	0.731	21.462	< 0.001^∗∗^	53.157	< 0.001^∗∗^
Beni Suef	123	30.8	106	26.5	171	42.8	110	27.5	89	22.3	201	50.2					4.627	0.099
Artificial intelligence allows patients to gain greater control over their health.																		
Ain Shams	119	30.8	114	29.5	153	39.6	57	14.8	78	20.2	251	65.0	1.713	0.425	32.432	< 0.001^∗∗^	52.363	< 0.001^∗∗^
Beni Suef	110	27.5	114	28.5	176	44.0	107	26.8	112	28.0	181	45.3					0.129	0.937
Artificial intelligence facilitates patient health education.																		
Ain Shams	114	29.5	115	29.8	157	40.7	75	19.4	54	14.0	257	66.6	1.319	0.517	29.032	< 0.001^∗∗^	54.220	< 0.001^∗∗^
Beni Suef	130	32.5	106	26.5	164	41.0	100	25.0	106	26.5	194	48.5					6.427	0.040^∗^
Artificial intelligence reduces human error in nursing practices and makes decisions more accurate.																		
Ain Shams	112	29.0	116	30.1	158	40.9	65	16.8	63	16.3	258	66.8	2.020	0.364	12.695	0.002^∗^	52.211	< 0.001^∗∗^
Beni Suef	106	26.5	139	34.8	155	38.8	88	22.0	94	23.5	218	54.5					21.002	< 0.001^∗∗^
Do you think artificial intelligence will replace doctors, pharmacists, or nurses in the healthcare system?																		
Ain Shams	113	29.3	117	30.3	156	40.4	80	20.7	58	15.0	248	64.2	1.823	0.402	29.819	< 0.001^∗∗^	46.484	< 0.001^∗∗^
Beni Suef	135	33.8	114	28.5	151	37.8	106	26.5	111	27.8	183	45.8					6.595	0.037^∗^
Artificial intelligence preserves patient data.																		
Ain Shams	108	28.0	125	32.4	153	39.6	60	15.5	76	19.7	250	64.8	3.917	0.141	12.183	0.002^∗^	49.007	< 0.001^∗∗^
Beni Suef	120	30.0	104	26.0	176	44.0	83	20.8	107	26.8	210	52.5					9.781	0.008^∗^

^∗^ *p* < 0.05.

^∗∗^ *p* < 0.001.

Table 4 demonstrates that a significant improvement in students’ mean knowledge score with 19.85 ± 2.14 in the Ain Shams student group compared with 17.49 ± 2.41 in the Beni Suef student group. Also, in relation to attitude mean score, the findings demonstrate 32.19 ± 2.77 in the Ain Shams student group compared with 29.23 ± 2.93 in the Beni Suef student group following the educational orientation session on AI with a statistical significant difference.

**TABLE 4:** Comparison of the total knowledge and attitude level within groups (pre‐ and postintervention) and between groups (Ain Shams vs. Beni Suef students).

	Preintervention						Postintervention						(*p*‐value) between groups		(*p*‐value) within group (pre vs. post)
	Low knowledge	Moderate knowledge			Good knowledge		Low knowledge	Moderate knowledge			Good knowledge		Pre vs pre	Post vs post	
	*n*	%	*n*	%	*N*	%	*N*	%	*n*	%	*n*	%			
Knowledge level															
Ain Shams	183	47.4	182	47.2	21	5.4	11	2.8	148	38.3	227	58.8	*X* ^2^ = 0.371, df = 2, *p* = 0.831	*X* ^2^ = 150.232, df = 2, *p* < 0.001^∗∗^	*X* ^2^ = 327.1107, df = 2, *p* < 0.001^∗∗^
Beni Suef	181	45.3	196	49.0	23	5.8	88	22.0	233	58.3	79	19.8			*X* ^2^ = 66.088, df = 2, *p* < 0.001^∗∗^
Knowledge score															
Ain Shams	15.70 ± 2.34						19.85 ± 2.14						*T* = 0.048, df = 784, *p* = 0.962	*T* = 14.537, df = 784, *p* < 0.001^∗∗^	*T* = 25.712, df = 385, *p* < 0.001^∗∗^
Beni Suef	15.71 ± 2.25						17.49 ± 2.41								*T* = 10.797, df = 399, *p* < 0.001^∗∗^

	**Negative attitude**			**Positive attitude**			**Negative attitude**			**Positive attitude**			**Pre vs pre**	**Post vs post**	
	***N***	**%**		***n***	**%**		***N***	**%**		***n***	**%**				

Attitude level															
Ain Shams	298	77.2		88	22.8		68	17.6		318	82.4		*X* ^2^ = 0.158, df = 1, *p* = 0.691	*X* ^2^ = 115.451, df = 1, *p* < 0.001^∗∗^	*X* ^2^ = 274.831, df = 1, *p* < 0.001^∗∗^
Beni Suef	304	76.0		96	24.0		218	54.5		182	45.5				*X* ^2^ = 40.772, df = 1, *p* < 0.001^∗∗^
Attitude score															
Ain Shams	27.27 ± 2.98						32.19 ± 2.77						*T* = 0.479, df = 784, *p* = 0.632	*T* = 14.543, df = 784, *p* < 0.001^∗∗^	*T* = 23.758, df = 385, *p* < 0.001^∗∗^
Beni Suef	27.38 ± 2.88						29.23 ± 2.93								*T* = 9.005, df = 399, *p* < 0.001^∗∗^

^∗^ *p* < 0.05.

^∗∗^ *p* < 0.001.

Figure **3** clearly shows an improvement in the students’ attitude levels following the implementation of the educational orientation session, as 82.4% of the Ain Shams student group demonstrated a positive attitude toward AI in the postorientation phase, compared to 22.8% before the educational guidelines were implemented. Similarly, 45.5% of the Beni Suef student group had a positive attitude postorientation, compared to 24% preimplementation.

**FIGURE 3:**
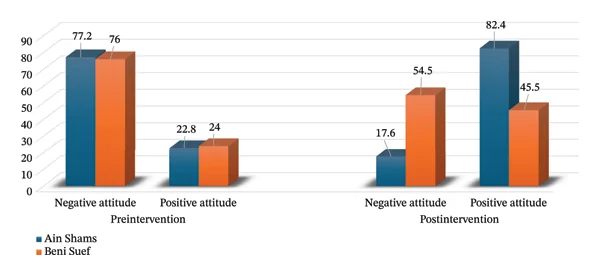
Comparison of students’ attitude levels toward artificial intelligence between Ain Shams and Beni Suef universities pre‐ and postintervention.

Table 5 shows that there is a significant correlation between knowledge and attitude improvement in the Ain Shams group after the educational orientation session compared with the Beni Suef student group.

**TABLE 5:** Correlation between total knowledge and attitude toward artificial intelligence in both groups.

	Knowledge			
	Ain Shams		Beni Suef	
	R	*p*	R	*p*
Attitude				
Preintervention	0.076	0.120	0.050	0.320
Postintervention	0.306	< 0.001^∗∗^	0.080	0.115

^∗^ *p* < 0.05.

^∗∗^ *p* < 0.001.

## 4. Discussion

The current study aimed to evaluate the effect of orientation sessions on maternity nursing students’ knowledge and attitudes toward the application of AI in obstetric nursing at Ain Shams and Beni Suef universities. The findings strongly support the achievement of this aim, as significant improvements were observed in both knowledge and attitudes following the intervention.

In relation to the first objective—assessing students’ initial levels of knowledge, the results revealed that the majority of students in both universities had low to moderate knowledge before the intervention, which aligns with previous literature, suggesting that 21 nursing students generally possess limited understanding of AI concepts [21, 22]. This confirms the necessity of introducing structured educational programs on AI within nursing curricula.

After implementing the orientation sessions, students demonstrated substantial improvement in all components of AI knowledge. This directly supports Hypothesis 1, which proposed that students would show a significant increase in knowledge following the sessions. The results of the current study are consistent with the findings of [23, 24], who reported that educational interventions significantly enhance healthcare students’ AI knowledge. The marked rise in correct answers across all knowledge domains indicates that the educational content was appropriate, clearly delivered, and effectively tailored to students’ learning needs.

Regarding the second objective—assessing attitudes toward AI—baseline results showed that many students had neutral or negative attitudes, possibly due to misconceptions, fear of replacement, or limited exposure to AI technologies. Such negative preconceptions have been documented in previous studies [25, 26]. After the intervention, students’ attitudes shifted markedly toward positivity, supporting Hypothesis 2 of the study. This shift reflects increased confidence, better understanding of AI’s potential, and reduced anxiety associated with technology use. These findings are in agreement with [27] who reported that improved knowledge significantly contributes to more favorable attitudes.

The results also revealed a statistically significant positive correlation between knowledge and attitude after the intervention, especially among Ain Shams students. This relationship reinforces the theoretical assumption that increased awareness drives acceptance—an observation well supported by earlier research [28].

The final objective was to compare the effect of the orientation sessions between the two universities, which directly relates to Hypothesis 3. While both groups showed significant improvements in knowledge and attitudes, Ain Shams students demonstrated notably higher postintervention knowledge scores. Similar institutional differences have been reported in studies conducted by [29] who emphasized that contextual factors can influence students’ digital readiness.

The findings of the present study demonstrated a significant improvement in students’ knowledge and attitudes toward AI in gynecology nursing following the educational intervention in both Ain Shams and Beni Suef universities. Before the intervention, most students in both groups showed low or moderate knowledge levels, while only a small proportion exhibited good knowledge. This aligns with several previous studies, reporting that nursing students generally possess limited baseline knowledge regarding AI applications in healthcare [21, 22].

After the intervention, there was a marked rise in the percentage of students achieving good knowledge levels, particularly at Ain Shams University. This improvement may be attributed to the structured and interactive educational sessions, which helped bridge the knowledge gap. Similar positive effects of educational interventions have been documented in studies conducted in Egypt and internationally, emphasizing that targeted training significantly enhances students’ understanding of AI concepts [23, 24].

Regarding attitudes, the study revealed that although most students initially exhibited negative or neutral attitudes toward AI, their attitudes shifted significantly toward the positive end after the intervention. This is consistent with the findings of [25] who stated that exposure to AI‐related education increases acceptance and decreases fear or misconceptions among nursing students. The increase in positive attitudes may also reflect improved confidence and awareness of the potential role of AI in supporting clinical decision‐making, reducing medical errors, and improving maternal–fetal outcomes.

The postintervention correlation analysis indicated a significant positive relationship between knowledge and attitude among Ain Shams students, suggesting that increased knowledge may contribute to more positive perceptions of AI. This aligns with the theory that familiarity enhances acceptance, as demonstrated in studies by [20, 26]who found that higher knowledge is associated with a greater willingness to integrate AI into nursing practice.

Moreover, the current study’s results showed a significant improvement regarding students’ perception of AI advantages, challenges, future roles, and potential applications in gynecology nursing. These findings are in line with research highlighting that nursing students recognize AI’s potential in patient assessment, emergency response, health education, and reducing human errors once they receive adequate training [27, 29].

Differences between the two universities postintervention may be explained by variations in previous exposure to digital tools, infrastructure, or students’ academic environment. Similar regional differences were reported by [28], indicating that institutional factors can influence the extent of improvement in digital literacy and AI readiness.

Overall, the results support the need for integrating AI education into nursing curricula to equip future nurses with essential competencies required in modern healthcare systems. This is reinforced by international recommendations emphasizing that AI literacy should be a core component in nursing education [30, 31].

From the researcher’s perspective, the findings highlight the crucial role of structured educational interventions in preparing maternity nursing students to effectively engage with AI technologies. The significant improvements observed postintervention suggest that the gap in students’ knowledge and attitudes is not rooted in resistance but rather in a lack of exposure. Once students receive well‐designed instructional content, they demonstrate enthusiasm, acceptance, and readiness to adopt AI as a supportive tool in obstetric nursing.

The researcher believes that integrating AI‐related content into maternity nursing curricula is no longer optional; it is essential for equipping future nurses with the skills required in modern healthcare. Furthermore, the differences observed between universities underscore the need for standardized national strategies that ensure equal access to technological education across institutions. Orientation sessions such as those conducted in this study can serve as a model for larger scale implementation.

## 5. Study Limitations and How They Were Addressed

Limitations were identified in this study. First, the use of a convenience sample drawn from only two universities (Ain Shams University and Beni Suef University) may limit the generalizability of the findings to other nursing students or educational settings. However, including students from two different institutions with varying contexts helped to enhance the diversity of the sample and partially improve external validity.

Second, the quasiexperimental design did not include a nonintervention control group, which limits the ability to attribute observed improvements solely to the orientation sessions. To mitigate this, a pre‐ to postassessment design was employed, allowing each participant to serve as their own control and enabling the measurement of changes following the intervention.

Third, the reliance on self‐reported questionnaires may introduce response bias. This limitation was addressed by ensuring anonymity and confidentiality through the use of coded questionnaires and by clearly informing participants that their responses would be used for research purposes only, thereby encouraging honest reporting.

Finally, the postintervention evaluation was conducted immediately after the orientation sessions, limiting the ability to assess long‐term retention and sustainability of the intervention effects. Nevertheless, this approach allowed for the accurate measurement of the immediate impact of the educational intervention. Future studies are recommended to use randomized controlled designs, more representative samples, and longitudinal follow‐up assessments to further strengthen the evidence.

## 6. Conclusion

The findings of the study supported all three research hypotheses. Following the orientation sessions, maternity nursing students showed a significant improvement in their knowledge levels regarding the application of AI in obstetric nursing, confirming the effectiveness of the intervention in enhancing knowledge. Additionally, students demonstrated a more positive attitude toward the use of AI in obstetric nursing, indicating that the sessions positively influenced their perceptions and acceptance of AI in practice. Furthermore, a significant difference was observed in the effect of the orientation sessions on knowledge and attitudes between students from Ain Shams University and Beni Suef University, suggesting that the intervention’s impact varied by study setting. Overall, these results highlight the importance of structured educational interventions in improving both knowledge and attitudes toward AI among maternity nursing students.

## 7. Recommendations

- Integration of AI Education into Nursing Curricula: Nursing programs, particularly in maternity and obstetric care, should incorporate structured content on AI to enhance students’ knowledge and readiness to apply AI technologies in clinical practice.
- Regular Educational Interventions: Orientation sessions, workshops, and seminars on AI applications should be conducted periodically to reinforce awareness and foster positive attitudes among nursing students.
- Tailored Approaches for Different Settings: Since the impact of interventions varied between universities, educational strategies should be adapted to the specific needs, resources, and learning environments of each institution to maximize effectiveness.
- Practical Application Opportunities: Nursing students should be provided with hands‐on experiences and case‐based learning involving AI tools in maternal and fetal care to bridge the gap between theory and practice.
- Ongoing Research and Evaluation: Further studies should be conducted to assess the long‐term effects of AI education on students’ competencies and to explore innovative methods for integrating AI in nursing practice.

## Author Contributions

Eman Said Abo El Ella Hassan contributed to the conceptualization and design of the study, data collection, data analysis, and preparation of the original manuscript draft. Eman Mostafa Sayed Ahmed contributed to the development of the methodology, supervision of the research process, interpretation of the findings, and critical revision of the manuscript. Somaia Ragab Eid participated in data collection, literature review, and drafting of the manuscript. Wafaa Gomaa Abdallah Eliewa contributed to data analysis, interpretation of the results, and editing of the manuscript. Samah Osman Ali was responsible for supervision, project administration, and final review and approval of the manuscript.

## Funding

No funding was received for this research.

## Disclosure

All authors read and approved the final version of the manuscript.

## Conflicts of Interest

The authors declare no conflicts of interest.

## Data Availability

The data that support the findings of this study are available from the corresponding author upon reasonable request.
